# Distribution and metabolism of iPSC-MSCs in the joint cavity of an osteoarthritis rat model

**DOI:** 10.3389/fbioe.2025.1555983

**Published:** 2025-06-17

**Authors:** Xiaoyang Yuan, Xulei Wang, Tianxi Du, Feng Zhang, Gang Cheng, Kang Wang, Haochen Xu, Pingting Yang, Yan Chang, Wei Wei, Peng He, Shangxue Yan

**Affiliations:** ^1^ Institute of Clinical Pharmacology, Anhui Medical University, Key Laboratory of Anti-inflammatory and Immune Medicine, Ministry of Education, Hefei, China; ^2^ Laboratory Animal Center, Anhui Medical University, Hefei, China; ^3^ Department of Orthopedics, First Affiliated Hospital of Anhui Medical University, Hefei, China

**Keywords:** iPSC-MSCs, Antares2-iMSCs, osteoarthritis, metabolism, biodistribution

## Abstract

**Introduction:**

To investigate the metabolism and distribution of iPSC‐MSCs in the joint cavity of rats with knee osteoarthritis (KOA).

**Methods:**

The iPSC‐MSCs labeled with the Antares2 luciferase gene were injected into the knee joints of rats, and then the metabolism and distribution of the cells in vivo were revealed by imaging and molecular biomarker methods.

**Result:**

Histopathological results demonstrated that iPSC‐MSCs significantly reversed joint tissue damage of arthritic rats. The fluorescence signal of iPSC‐MSCs labeled with Antares2 luciferase gene was stable and persistent with high detection sensitivity. The fluorescent signal duration of Antares2‐iMSCs in the joint cavity of KOA rats was approximately 2 weeks, which was significantly longer than 1 week in the sham‐operated group. The proportion of iPSC‐MSCs in the synovial fluid gradually decreased over time, and for the first time, the cells were observed to attach to the synovium first, followed by the meniscus and cartilage.

**Discussion:**

This study was the first to explore the metabolism and distribution of iPSC‐MSCs after intra‐articular injection by labeling the Antares2 luciferase gene, which provides assurance and theoretical basis for the safety of clinical application of iPSC‐MSCs in treating osteoarthritis.

## 1 Introduction

Osteoarthritis (OA) is the most common joint disease worldwide, affecting approximately 10% of men and 18% of women over 60 years of age (Cao et al., 2024). There are multiple factors that contribute to the occurrence and progression of OA. It is characterized by gradual degeneration and exfoliation of the articular cartilage, as well as structural and functional changes in the joint as a whole, including the synovium, meniscus, periarticular ligaments, and subchondral bone (Ebada et al., 2022; Mobasheri and Batt, 2016). Currently, there are four main treatments for OA: physical therapy, such as weight loss and reducing joint damage; using non-steroidal anti-inflammatory drugs (NSAIDs) to relieve the patient’s pain; intra-articular (IA) injections of drugs such as sodium hyaluronate; and joint replacement surgery for patients with severe or advanced OA (Huang et al., 2022). However, current treatments are aimed at relieving patient symptoms such as localized swelling and pain, which do not effectively improve cartilage damage (Hochberg et al., 2012). In the past decade, cell therapy for OA has developed rapidly, in which stem cell therapy has achieved excellent results (Ruscitto et al., 2023; Song et al., 2020).

Mesenchymal stem cells (MSCs) have a strong differentiation potential, can be isolated and extracted from a variety of autologous and allogeneic sites such as bone marrow (BMMSCs), adipose tissue (ADMSC), umbilical cord blood and dental pulp. In fact, there have been relevant reports about clinical trials of bone marrow MSCs, adipose MSCs, and umbilical cord MSCs for the treatment of OA (Epanomeritakis and Khan, 2024; Matas et al., 2019; Shariatzadeh et al., 2019). However, there are still many difficulties to overcome in stem cell transplantation therapy, including the risk of tumor formation, ethical issues, and transplant rejection. iPSC-MSCs are constructed by transferring transcription factors such as Oct3/4, Sox2, c-Myc and Klf4 into adult cells via lentiviral vectors, which transform them into pluripotent stem cells with embryonic stem cell-like functions (Takahashi and Yamanaka, 2006; Yu et al., 2007). It has the advantages of low immunogenicity and high proliferative capacity compared to other sources of MSCs. Our pre-experiment confirmed that iPSC-MSCs were used to treat a rat OA model and achieved favorable results, and the pathological analysis showed that it could repair cartilage damage and regenerate damaged cartilage. However, we are still unknown about the fate of iPSC-MSCs after injection into the joint cavity of osteoarthritis, including cell viability, cell distribution location, attachment tissue, and intra-articular retention period. The exploration of the pharmacokinetics of iPSC-MSCs after knee injections is essential for adjusting the administration regimen, understanding the pattern of cellular effects, improving the formulation, and improving therapeutic efficacy.

It has been demonstrated that after MSCs with the luciferase gene were injected into the joint cavity, fluorescent signals could be observed at different time points. Moreover, the cells in the osteoarthritis group survived longer than those in the control group, suggesting that different joint cavity microenvironments affect cell metabolism and proliferation (Li et al., 2024). There is also direct evidence suggesting that MSCs are still acting locally, but their distribution within the joint cavity is unknown (Li et al., 2016). *In vivo* biofluorescence imaging refers to the utilization of luciferase genes expressed in animals to produce luciferase protease that reacts with the corresponding substrate to generate a light signal and then forms an image *in vitro* through sensitive charge-coupled device equipment (Yeh et al., 2017). The current cell labeling methods have drawbacks such as low sensitivity, low specificity, and dependence on gene-edited animals (He et al., 2023). Antares2 is an excellent bioluminescent reporter gene with a stable and long-lasting fluorescent signal, favorable biosafety, and high detection sensitivity (Hikita et al., 2020; Yeh et al., 2017). Currently, no relevant studies use the Antares2 luciferase reporter gene to observe the metabolic and distributional characteristics of iPSC-MSCs in rats.

In this study, we injected Antares2-iMSCs into the knee joints of non-transgenic rats and then explored the metabolism and distribution of the cells by *in vivo* fluorescence imaging. Our previous work confirmed that iPSC-MSCs had a favorable therapeutic effect on OA rats by pathological analysis including, HE staining and immunohistochemistry. Exploring the distribution and metabolic change patterns of Antares2-iMSCs in different joint microenvironments is conducive to further optimizing stem cell therapy protocols, which provides the experimental basis for applying iPSC-MSCs in the clinical treatment of OA.

## 2 Materials and methods

### 2.1 Cell culture

The iPSC-MSCs (Lot No.: 202011006) and Antares2-iMSCs (Lot No.: 20201206) were purchased from Nuwacell Biotechnology Co (Hefei, China). The above cells were cultured in MEM-α medium containing 10% fetal bovine serum (FBS). MEM-α medium was obtained from Procell Life Science & Technology Co (Wuhan, China). FBS was purchased from Biological Industries^®^ (Kibbutz Beit Haemek, Israel).

### 2.2 Animals

Specific pathogen-free (SPF)-grade SD rats (weighing about 250 g, male) were purchased from the SiBeiFu Biotechnology Co., Ltd. (Beijing, China) (Certification: SXK (Beijing) 2019-0010). The animals are fed in an SPF-grade environment with constant temperature (25°C ± 2°C) and constant humidity (70% ± 5%). The relevant operations in this study were carried out following relevant guidelines for animal experiments and were approved by the Animal Research Ethics Committee of the Institute of Clinical Pharmacology of Anhui Medical University.

### 2.3 Establishment of rat osteoarthritis model

The operation was performed in a sterile SPF animal laboratory. First, the rats were anaesthetised with Zoletil 100 (100 g/0.1 mL) (Virbac, France). The left hind limb of the rats was shaved and disinfected with 75% alcohol, and then a 20 mm incision was made to expose the left knee joint. The medial collateral ligament and the anterior cruciate ligament were cut after exposing the rat knee joint, and then the medial meniscus of the knee joint was excised. However, the rats in the sham operation group did not destroy any ligaments or menisci after exposing the joint cavity.

### 2.4 Cell therapy grouping and administration

Forty rats were randomly divided into four groups (n = 10): sham operation group (single intra-articular injection of 50 μL saline), model group (single intra-articular injection of 50 μL saline), iPSC-MSCs group (single intra-articular injection of 50 μL saline containing 2.0 × 10^6^ cells), and sodium hyaluronate (SH) group (intra-articular injection of 50 μL sodium hyaluronate containing 0.5 mg; weekly; five times).

### 2.5 Histopathological studies

At the end of the experiment, the knee joints of the rats were collected for histopathological evaluation. Tissue sections of the knee joint were stained by hematoxylin and eosin to observe inflammatory cells, synovial infiltration, and cartilage damage. To assess the articular cartilage damage, the tissue sections of the knee joint were immunostained with antibodies against MMP-13 (diluted 1:200, ab219620, Abcam, United Kingdom), OPG (diluted 1:200, ab203061, Abcam, United Kingdom), type I collagen (diluted 1:200, abs120555, Absin, Shanghai, China), and type II collagen (diluted 1:100, ab34712, Abcam, United Kingdom), respectively.

### 2.6 Analysis of cell proliferation

The MSCs (2.5 × 10^4^ cells/well) were seeded into 24-well plates and cultured for 48 h. Then the cells were washed three times with phosphate buffer solution (PBS) and fixed with 4% paraformaldehyde for 30 min. The nuclei were stained with DAPI (Solarbio, Beijing, China) for 3 min. The number of MSCs in each group was calculated by high-content imaging to determine the value-added of Antares2-labeled MSCs and unlabeled MSCs.

### 2.7 *In vitro* and *in vivo* biofluorescent imaging

To determine the *in vitro* detection threshold, a 100 μL PBS suspension containing Antares2-iMSCs (10^6^, 10^5^, 10^4^, 10^3^, 0 cells) was first prepared. Then, 100 μL of the configured Diphenylterazine (DTZ, 2 mg/mL, MCE, China) solution was added to each EP tube and mixed well. At the same time, the same dose of Antares2-iMSCs was injected into the knee joint cavity of the rats, and then the configured 100 μL of DTZ solution (2 mg/mL) was injected into the rats through the tail vein. The rats were immediately scanned by a small animal live imager (Amix, Spectral Instruments Imaging, United States) to determine the detection threshold *in vivo*. The same method was used to investigate the metabolism profile of Antares2-iMSCs in the knee joint at different time periods.

### 2.8 Metabolic distribution study grouping and administration

After 8 weeks of modelling, the Antares2-iMSCs were injected into the knee joint cavity of rats. The rat knee joint was injected with 50 μL of saline containing 2.0 × 10^6^ cells. The rats were then randomly divided into six groups (n = 5): stem cell injection for 5 h; stem cell injection for 24 h; stem cell injection for 72 h; stem cell injection for 1 week; stem cell injection for 2 weeks; and stem cell injection for 6 weeks.

### 2.9 Biofluorescence imaging of joint tissue from rats

The synovium, meniscus, and cartilage tissues were taken from the joint cavity and placed in a Petri dish after euthanising the rats. Subsequently, 50 μL of DTZ (2 mg/mL) solution was added dropwise to the joint tissue, and the DTZ solution was removed from the tissue after 30 s. Finally, fluorescence imaging of joint tissue was performed using a small animal imaging system.

### 2.10 Detection of CD90 expression in different tissues by immunofluorescence

The synovium, cartilage and meniscus embedded in the OCT were sliced into 3.5um thick sections using a frozen microtome (CM1950, Leica, Germany). To assess the expression of CD90 in tissues, the tissue sections were immunofluorescence stained with an antibody against CD90 (diluted 1:100, ab92574, Abcam, United Kingdom).

### 2.11 Detection of CD90 expression in synovial fluid by flow cytometry

The synovial fluid was filtered with a sieve (200 mesh) to obtain a single-cell suspension. Subsequently, the cells were washed twice with PBS and stained with FITC-CD90 antibody (BD Pharmingen, United States). Finally, the fluorescence signal of each group was detected by flow cytometry (BD FACSC anto, BD Biosciences, United States).

### 2.12 Statistical analysis

Data were statistically analyzed by statistical program SPSS version 22.0 (SPSS Inc., Chicago, IL). The comparison between the multiple groups uses one-way ANOVA, followed by Dunnett’s test to detect intergroup differences. The data were presented as the mean ± SD. P-value <0.05 was considered statistically significant.

## 3 Results

### 3.1 The therapeutic efficacy of iPSC-MSCs for OA

**FIGURE 1:**
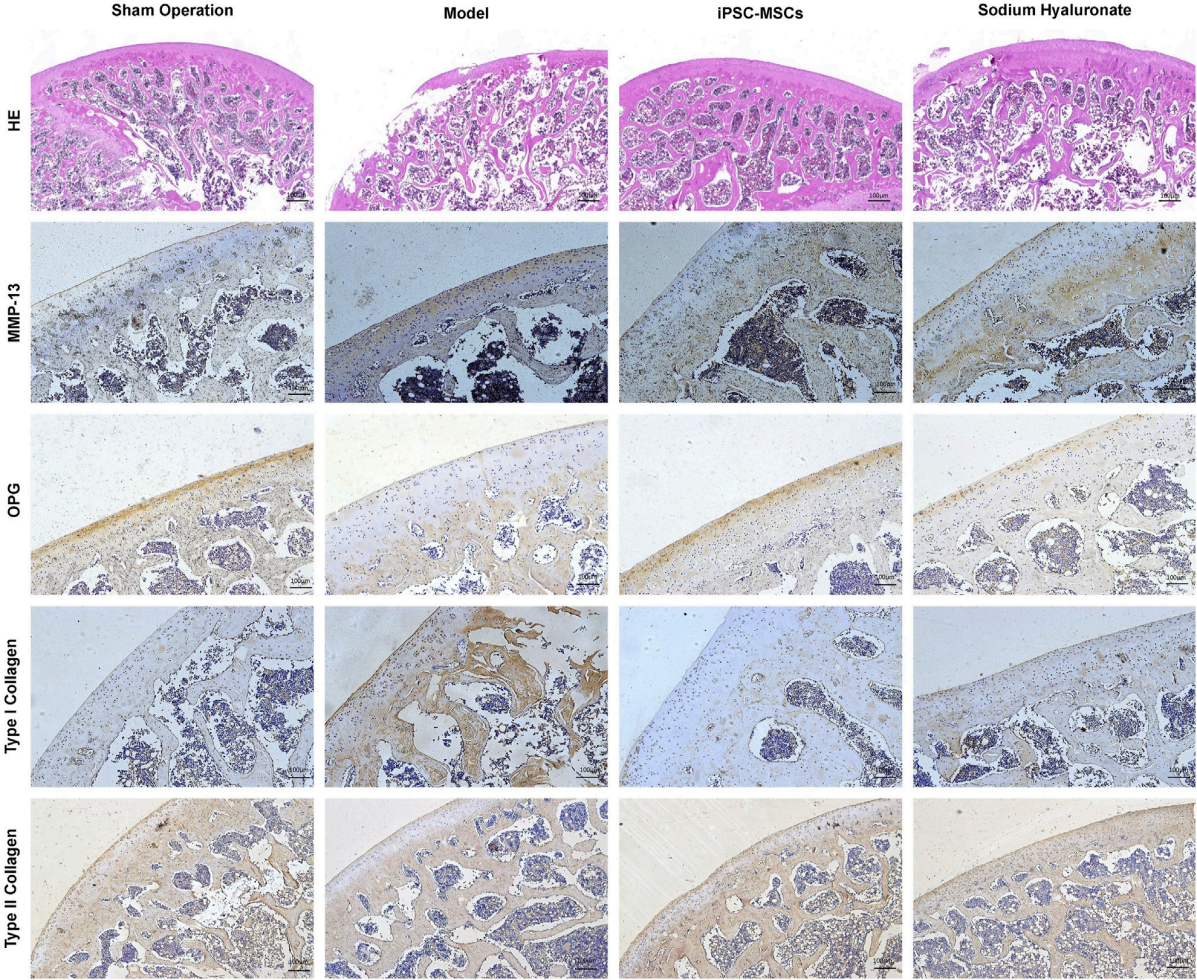
HE and immunohistochemical analyses of joint slices from different groups to evaluate the therapeutic effect of iPSC-MSCs.

Pathological changes of OA in rats were investigated by HE staining. Compared with the sham-operated group, the pathology of the knee in the model group showed the typical changes of OA, including severe cartilage destruction, disturbed chondrocyte arrangement and synovial infiltration into the cartilage layer (Figure 1). However, treatment with iPSC-MSCs significantly increased the thickness of the articular cartilage layer, increased the number of chondrocytes, and decreased inflammatory cell infiltration in the joint cavity. Compared with the sham-operated group, cartilage tissue in the model group showed remarkably higher expression of MMP-13 and type I collagen, whereas osteoprotegerin (OPG) and type II collagen were significantly reduced (Figure 1). In contrast, iPSC-MSCs reversed the above alterations, with expression levels close to those of the sham-operated group.

### 3.2 The influence of the Antares2 luciferase gene on the proliferation of iPSC-MSCs

Whether the Antares2 luciferase gene affects the proliferation of iPSC-MSCs was verified by measuring the mean value of cells per unit area in each group with a high-content imaging system. The results showed that the proliferation rates of Antares2-iMSCs cultured for 24, 48 and 72 h had no statistical difference from that of iPSC-MSCs (Supplementary Figure S1).

### 3.3 Detecting the sensitivity of Antares2-iMSCs for biofluorescence imaging

**FIGURE 2:**
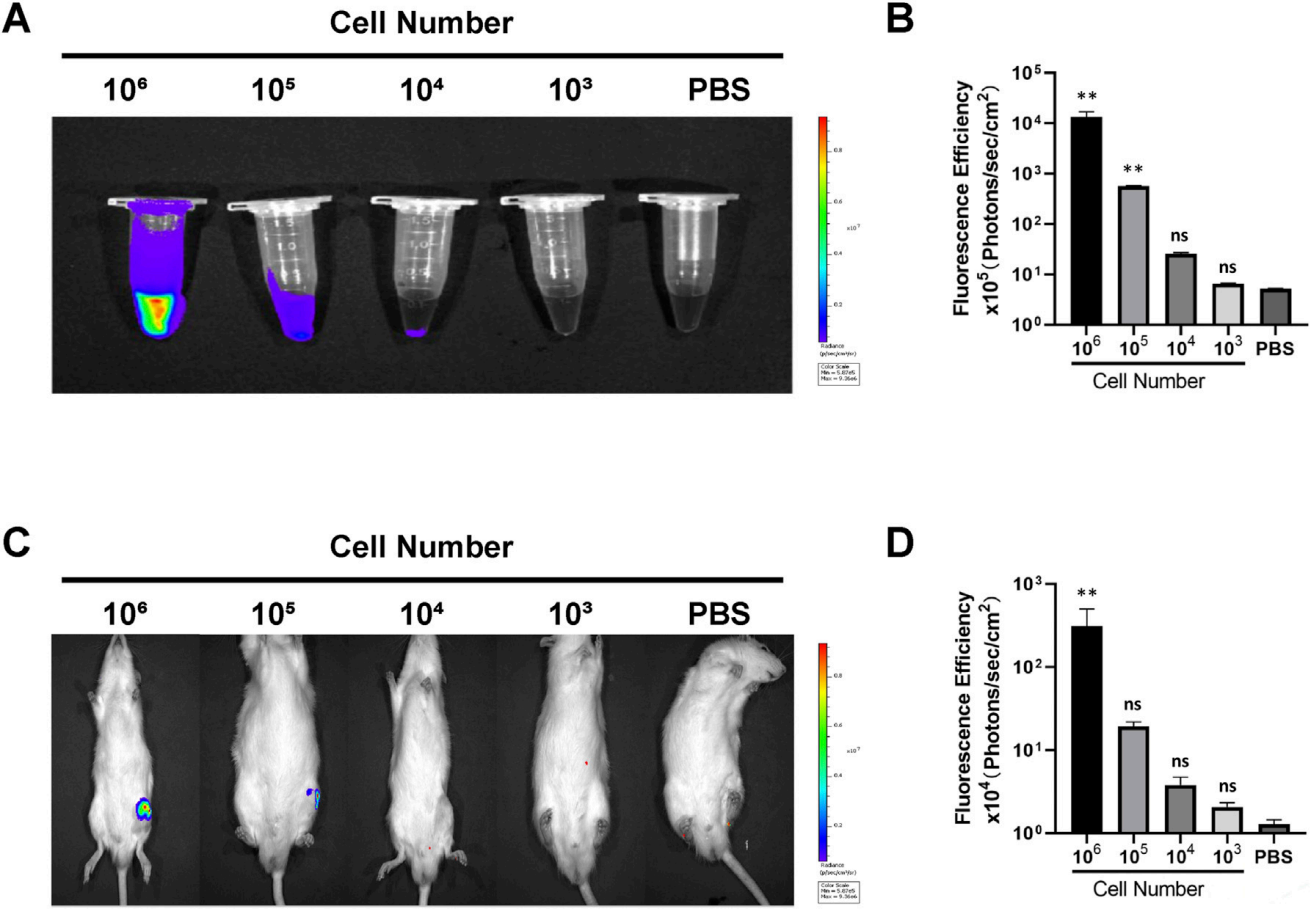
Detecting the tracking sensitivity of biofluorescence imaging. **(A)** The fluorescent signal in serial cell dilutions. **(B)** The results of quantitative analysis of cellular fluorescence signals *in vitro*. **(C)** The fluorescence signals of rats injected with serial cell dilutions. **(D)** The results of quantitative analysis of fluorescence signals *in vivo*. **P < 0.01.

The fluorescence intensity signal was positively correlated with the number of Antares2-iMSCs. The results showed that EP tubes containing 10^6^ and 10^5^ Antares2-iMSCs showed distinct strong fluorescence, whereas EP tubes with 10^4^ Antares2-iMSCs showed detectable marginal fluorescence (Figures 2A,B). No significant fluorescent signal could be detected in EP tubes containing 10^3^ Antares2-iMSCs, suggesting that the *in vitro* detection threshold of the IVIS Spectrum system for Antares2-iMSCs is between 10^4^ and 10^3^ cells.

After determining the sensitivity of the *in vitro* assay, the *in vivo* noninvasive detection threshold was explored by injecting Antares2-iMSCs into the knee joint of the rat. The results showed that rats injected with 10^6^ and 10^5^ Antares2-iMSCs displayed significant fluorescence signals, whereas rats injected with 10^4^ cells had no detectable signals, suggesting that the *in vivo* detection threshold of the IVIS Spectrum system for Antares2-iMSCs lies between 10^5^ and 10^4^ cells (Figures 2C,D).

### 3.4 The metabolism of Antares2-iMSCs after injection into the rat knee joint cavity

**FIGURE 3:**
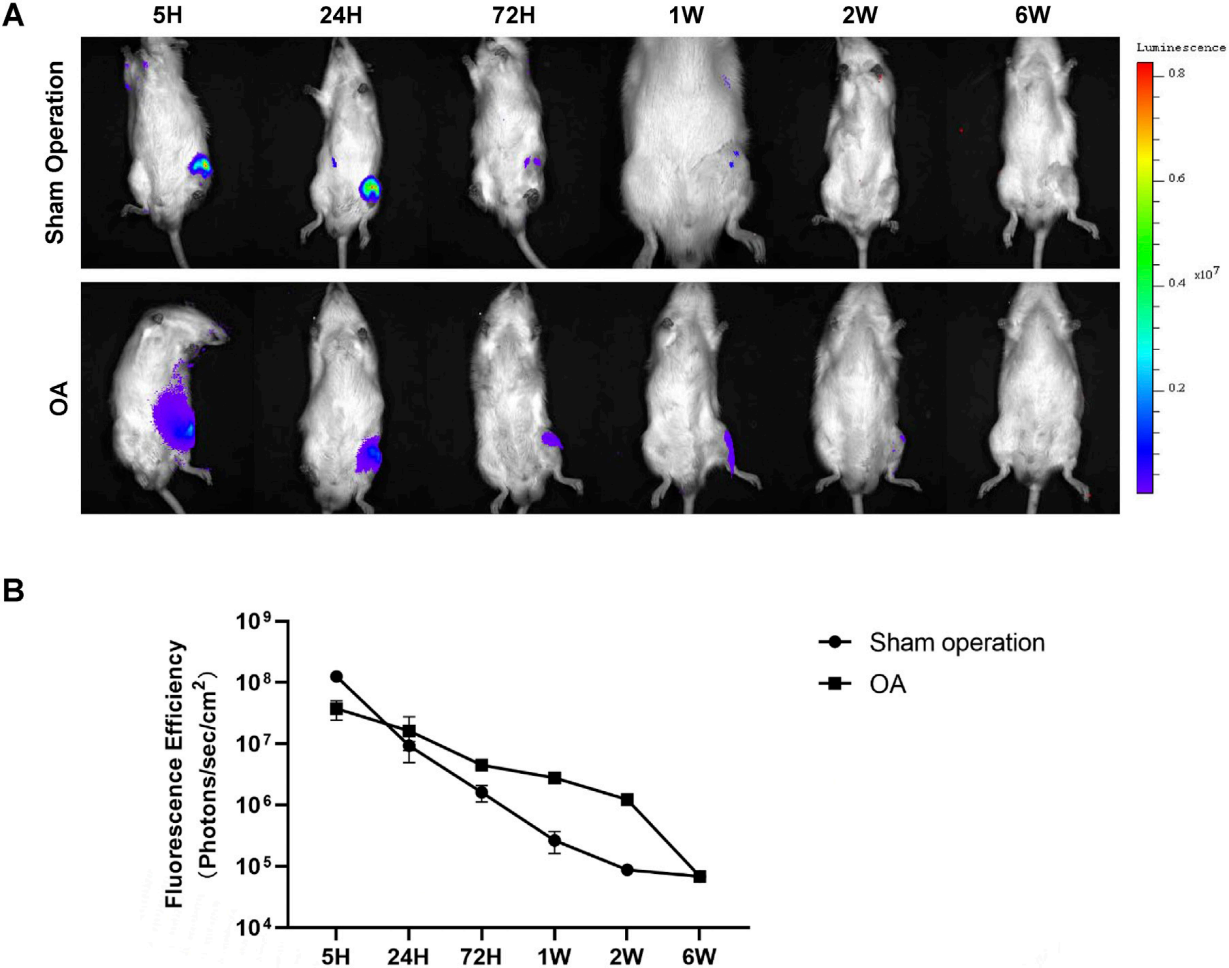
The metabolism of Antares2-iMSCs in the rat knee joint cavity. **(A)** The fluorescence signals were dynamically recorded in the sham-operated rats and the OA rats. **(B)** Quantified fluorescence intensity of the sham-operated and OA groups. (Values are presented as means ± sd.).

After determining the detection threshold, our study further explored the metabolic characteristics of Antares2-iMSCs at therapeutic doses in the knee joint. Firstly, 2 × 10^6^ Antares2-iMSCs were injected into the left knee joints of rats in the sham-operated group and the OA group, and the results showed that the fluorescence intensity was the strongest in the knee joints of the rats after 5 h and gradually decreased over time (Figure 3A). The results showed that the fluorescent signals disappeared in the sham-operated group 2 weeks after cell injection; however, the fluorescent signals of Antares2-iMSCs lasted longer in the model group of rats than in the sham-operated group (Figure 3B). The fluorescent signals detected in both groups were located near the knee joint cavity and did not migrate to other sites in the body (Figure 3A).

### 3.5 The distribution of iPSC-MSCs in joint tissues

**FIGURE 4:**
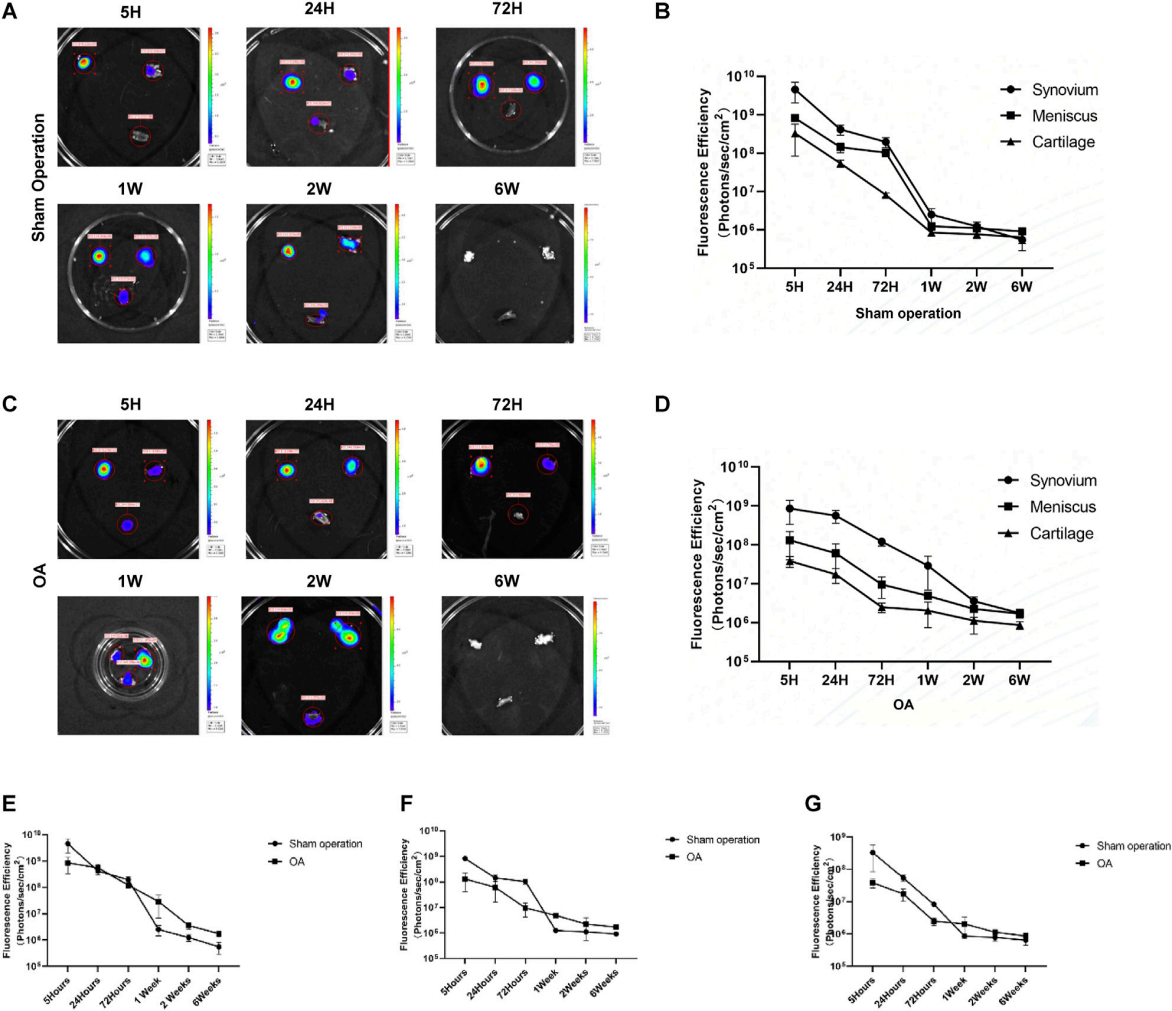
Tracking the distribution of iPSC-MSCs in joints. **(A)** Fluorescence imaging of synovial, meniscal and cartilage tissues in the joint cavity of rats in the sham-operation group. **(B)** Fluorescence intensity of synovium, meniscus and cartilage tissue in the sham-operation group. **(C)** Fluorescence imaging of synovial, meniscus and cartilage tissues in the joint cavity of rats in the OA group. **(D)** Fluorescence intensity of synovium, meniscus and cartilage tissue in the OA group. **(E–G)** Fluorescence intensity of synovial **(E)**, meniscal **(F)** and cartilage **(G)** tissues in the sham-operated and OA groups.

After euthanasia of the rats, the synovial, meniscus and cartilage tissues were removed from the joint cavity, and then the distribution of Antares2-iMSCs in each tissue was detected by fluorescence imaging. The results showed that the fluorescence signal in synovial, meniscal and cartilage tissues of the sham-operated and OA groups was strongest at 5 h and diminished over time (Figures 4B,D). The overall trend of decreasing fluorescence intensity was smoother in the OA group compared to the sham-operated group. After 1 week, the fluorescence signals of all three tissues in the OA group were higher than those in the sham-operated group (Figures 4E–G). There was the highest fluorescence intensity in the synovial membrane of both the sham-operated group and the OA group, followed by the meniscus and cartilage (Figures 4B,D). In addition, the fluorescence intensity of the three tissues decreased to similar levels at 1 week and 6 weeks in the sham-operated and OA groups, respectively.

### 3.6 The level of CD90 expression of iPSC-MSCs in joint tissues

**FIGURE 5:**
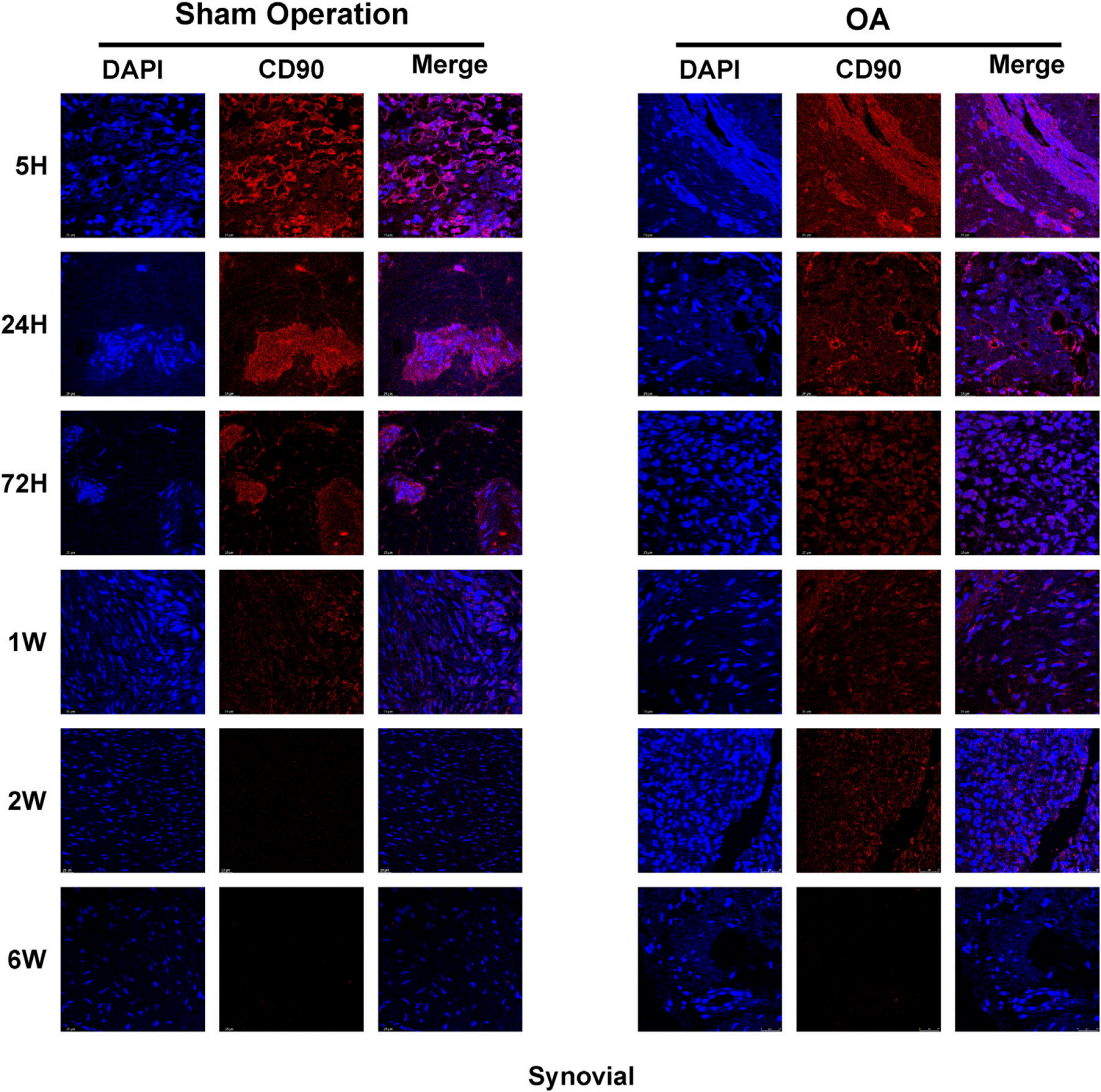
The expression of CD90/Thy protein in the synovium of rat joint cavities.

In addition to the initial investigation of the distribution of iPSC-MSCs in joint tissues by a biofluorescence imaging system, this study further used immunofluorescence to detect the expression of CD90 on synovium, cartilage and meniscus. The results showed that the expression of CD90 in the synovium, cartilage and meniscus of the sham-operated and OA groups was strongest at 5 h, and the expression of CD90 in various tissues decreased significantly with time (Figure 5; Supplementary Figures S2, S3). The decreasing trend of CD90 expression in the sham-operated and OA groups was similar to the results of fluorescence intensity detection.

### 3.7 Detection of CD90 expression level in synovial fluid by flow cytometry

**FIGURE 6:**
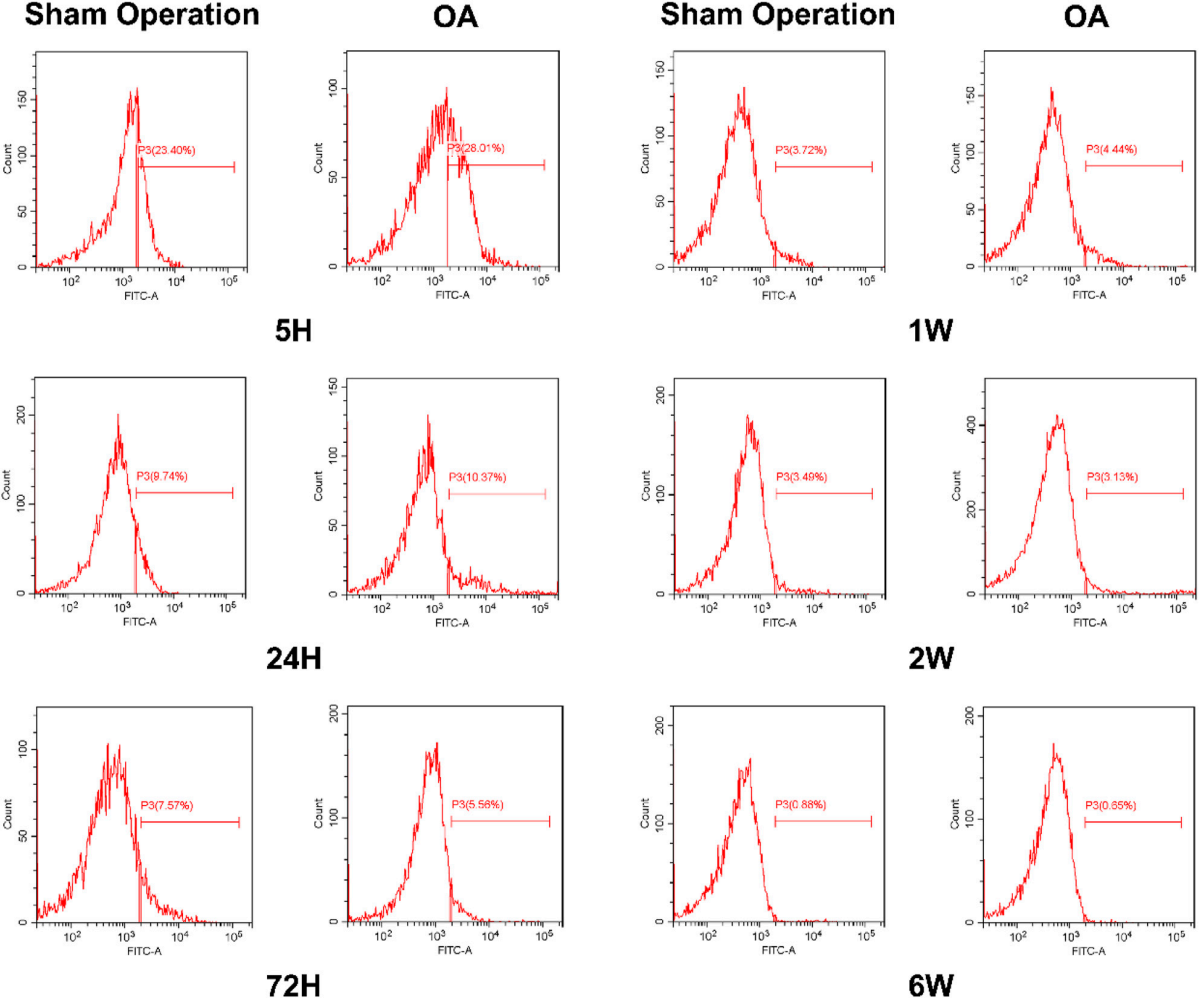
Detection of CD90 (iPSC-MSCs) expression levels in synovial fluid by flow cytometry.

To explore the number of iPSC-MSCs in the synovial fluid of the joint cavity, we examined the expression of the MSCs marker (CD90) at different time points using flow cytometry. The expression of CD90 in the synovial fluid of the sham-operated and OA groups was highest at 5 h and gradually decreased over time, with the greatest decrease between 5 and 24 h (Supplementary Figure S4). The expression of CD90 in synovial fluid of the OA group (25.18% ± 6.42%) was higher than that of the sham-operated group (20.79% ± 1.87%) at 5 h (Figure 6).

## 4 Discussion

Many previous studies have shown that intra-articular injection of MSCs has promising efficacy in the treatment of OA (Lamo-Espinosa et al., 2020; Lamo-Espinosa et al., 2018; Lin et al., 2024). Our experimental results suggest that treatment with iPSC-MSCs can significantly improve cartilage damage (Figure 1). In addition, iPSC-MSCs could reduce the expression of MMP-13 and elevate the expression of OPG in the knee joint, which could inhibit cartilage degradation and slow down the progression of OA (Abshirini et al., 2021). The expression of type I collagen was significantly elevated in the articular cartilage of rats in the OA group. iPSC-MSCs administration elevated the expression of type II collagen in the cartilage of rats and promoted the recovery of cartilage matrix. As an alternative source of stem cells, iPSC-MSCs can be induced from patient-specific adult cells and have similar characteristics to embryonic stem cells in terms of morphology, self-renewal and differentiation ability (Yu et al., 2007). In addition, iPSC-MSCs have superiority over other sources of MSCs in terms of cell proliferation, immunomodulation, production of exosomes with regulatory functions, and secretion of biologically active cytokines (Abe et al., 2023; Rosochowicz et al., 2024). However, the distribution and metabolism of iPSC-MSCs in the joint cavity after injection have not yet been reported.

In this study, Antares2 luciferase gene-labelled iPSC-MSCs were used as labelled cells, and DTZ was used as a luciferase substrate to study the metabolism and distribution of iPSC-MSCs in rats. Antares2, a BRET (bioluminescence resonance energy transfer)-based reporter, was created by inserting the fluorescent protein CyOFP1 into teLuc, which further improves bioluminescence detection in deep tissues (Yeh et al., 2017). Antares2-iMSCs as a new labelling method can be observed with strong fluorescence signals in the 695 ± 50 nm band by small animal *in vivo* imagers, which has the advantages of stable fluorescence signals and avoids spontaneous fluorescence in animals. Excitation of the external light source avoids the interference of the background signal from natural fluorescent substances, and possesses the characteristics of high specificity, excellent signal-to-noise ratio and extremely high sensitivity (Li et al., 2016). In addition, the results of the high-content proliferation assay showed that there was no significant difference in the proliferation of Antares2-iMSCs and iPSC-MSCs cultured for different time periods, suggesting that Antares2 labelling had no effect on the cellular activity of iPSC-MSCs (Supplementary Figure S1). Similarly, Antares2-iMSCs have a high detection sensitivity because the minimum detectable cell concentration is approximately 10^4^ cells *in vitro* and 10^5^ cells *in vivo* (Figure 2).

It has been shown that MSCs maintain tissue homeostasis and repair through replacement of mature cells lost due to physiological renewal, aging, and injury (Wu et al., 2024). The fate of MSCs in the knee joint is still unknown, and we need to understand the survival time and distribution characteristics of MSCs in the joint. In this way, we can explore their therapeutic mechanisms. The experimental results showed that iPSC-MSCs persisted in the joint cavity over 2 weeks after local injection and did not migrate to other sites, providing direct evidence that iPSC-MSCs exert therapeutic effects and carry out tissue repair locally rather than systemically (Figure 3A). Compared with the sham-operated group, the fluorescence signals of Antares2-iMSCs lasted longer in the rats from the OA group, which may be related to the inflammatory microenvironment of OA. The cytokines secreted in the inflammatory microenvironment of OA can attract MSCs to homing and staying locally for longer time (Fan et al., 2023).

Related studies have reported on the metabolism of synovial-derived MSCs in a knee meniscectomy model and showed that more synovial MSCs were attached to the meniscus in the model group compared to the control group (Baboolal et al., 2018). Therefore, we observed the distribution of iPSC-MSCs on tissues such as synovium, meniscus and cartilage in the joint cavity by fluorescence imaging and immunofluorescence (Figures 4, 5). The experimental results showed that iPSC-MSCs injected into the joint cavity were predominantly distributed in the synovium, followed by the meniscus and cartilage, and the cells were still detectable in the tissue until 6 weeks. According to our analysis, when cells are injected into the joint cavity they are first adsorbed onto the synovium, which consists of loose connective tissue, while the smooth meniscus and cartilage are difficult to adsorb. Therefore, the idea that intra-articularly injected MSCs exert a therapeutic effect by adsorbing to the damaged area and directly differentiating into new tissue needs further validation. In addition, it has been reported in the literature that synovial fluid has a complex composition containing a variety of inflammatory factors and chemokines (Ragni et al., 2022). MSCs can effectively reduce the level of pro-inflammatory monocytes/macrophages in synovial fluid (Chahal et al., 2019). Positive CD90 expression is the minimum criterion for MSC identification as defined by the International Society for Cellular Therapy, so we examined the level of CD90 expression in synovial fluid. The results showed the largest decrease of CD90 expression between 5 and 24 h, which is consistent with the results of *in vivo* fluorescence imaging (Supplementary Figure S4).

## 5 Conclusion

Although MSCs have shown significant efficacy in preclinical animal models, due to factors such as individual differences among patients and a certain proportion of non-response phenomena, MSCs therapy still faces many challenges in the process of clinical transformation. In this study, a novel cell labeling method was adopted to explore the metabolism and distribution of iPSC-MSCs after intra-articular injection. The results showed that the fluorescence signal duration of Antares2-iMSCs in the OA group exceeded 2 weeks, which was longer than that in the sham operation group. Moreover, stem cell administration fully alleviated the phenomenon of articular cartilage loss in OA rats; Intra-articular injection of iPSC-MSCs is widely distributed in the synovium, cartilage, meniscus and synovial fluid, among which the proportion contained in the synovium is the largest. Exploring the metabolic and distribution characteristics of iPSC-MSCs is helpful for guiding the administration frequency and understanding its mechanism of action. Therefore, Antares2 is an excellent bioluminescence reporter gene. This study further demonstrated the safety and feasibility of intra-articular injection of iPSC-MSCs in the treatment of OA.

## Data Availability

The original contributions presented in the study are included in the article/Supplementary Material, further inquiries can be directed to the corresponding authors.

## Supplementary material

SUPPLEMENTARY FIGURE S1 Effect of Antares2 luciferase gene on cell proliferation of iPSC-MSCs detected by high content cell imaging system.

SUPPLEMENTARY FIGURE S2 The expression of CD90/Thy protein in the meniscus of rat joint cavities.

SUPPLEMENTARY FIGURE S3 The expression of CD90/Thy protein in the cartilage of rat joint cavities.

SUPPLEMENTARY FIGURE S4 Fluorescence intensity of CD90 expression in rat synovial fluid.
