# Supplementary figures and images for: Distribution and metabolism of iPSC-MSCs in the joint cavity of an osteoarthritis rat model
Source: Front Bioeng Biotechnol. 2025 Jun 17;13:1555983. doi: 10.3389/fbioe.2025.1555983 (PMC12209229; doi:10.3389/fbioe.2025.1555983)

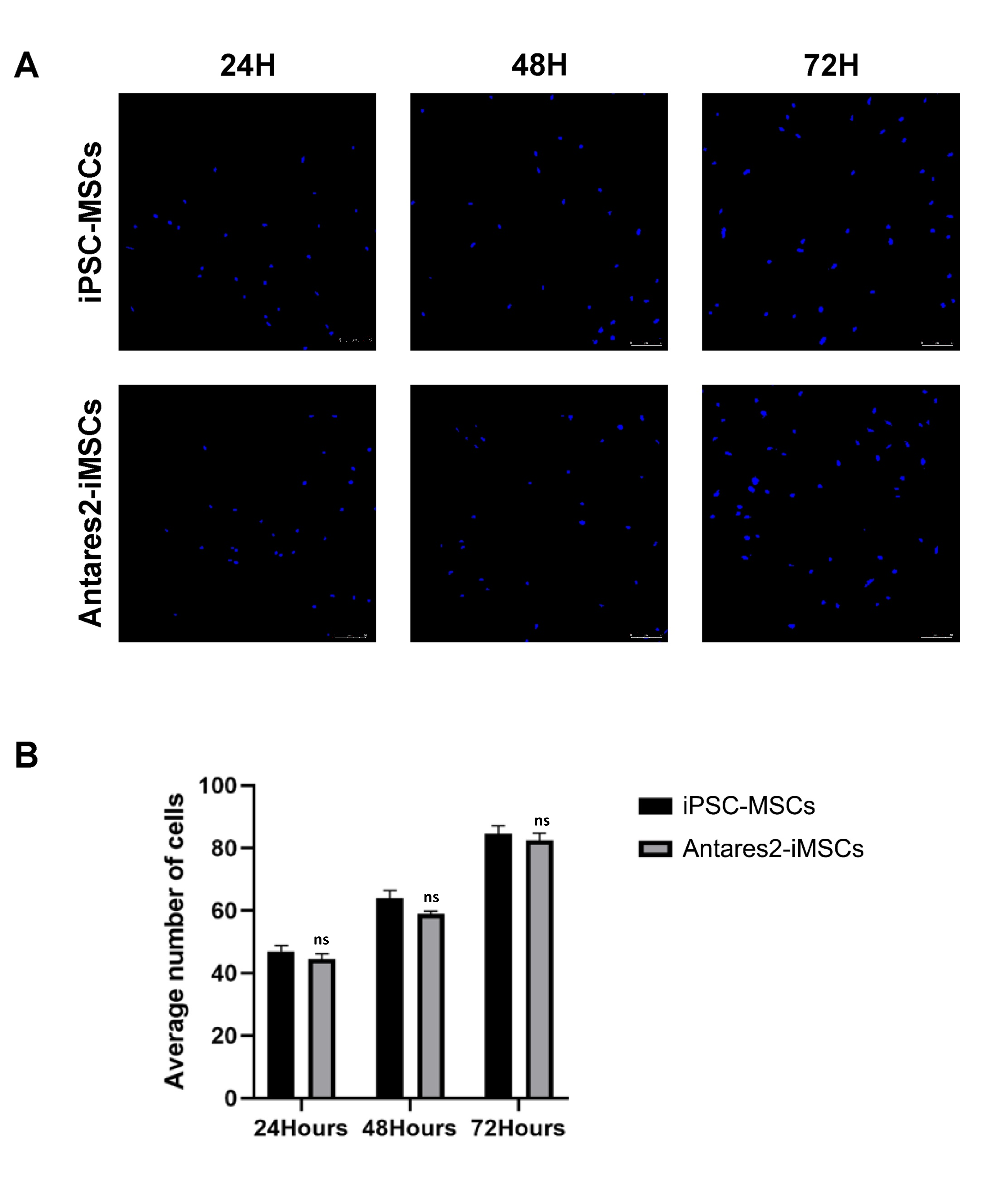

Supplement: Supplementary file 1 [file Image3.tif]

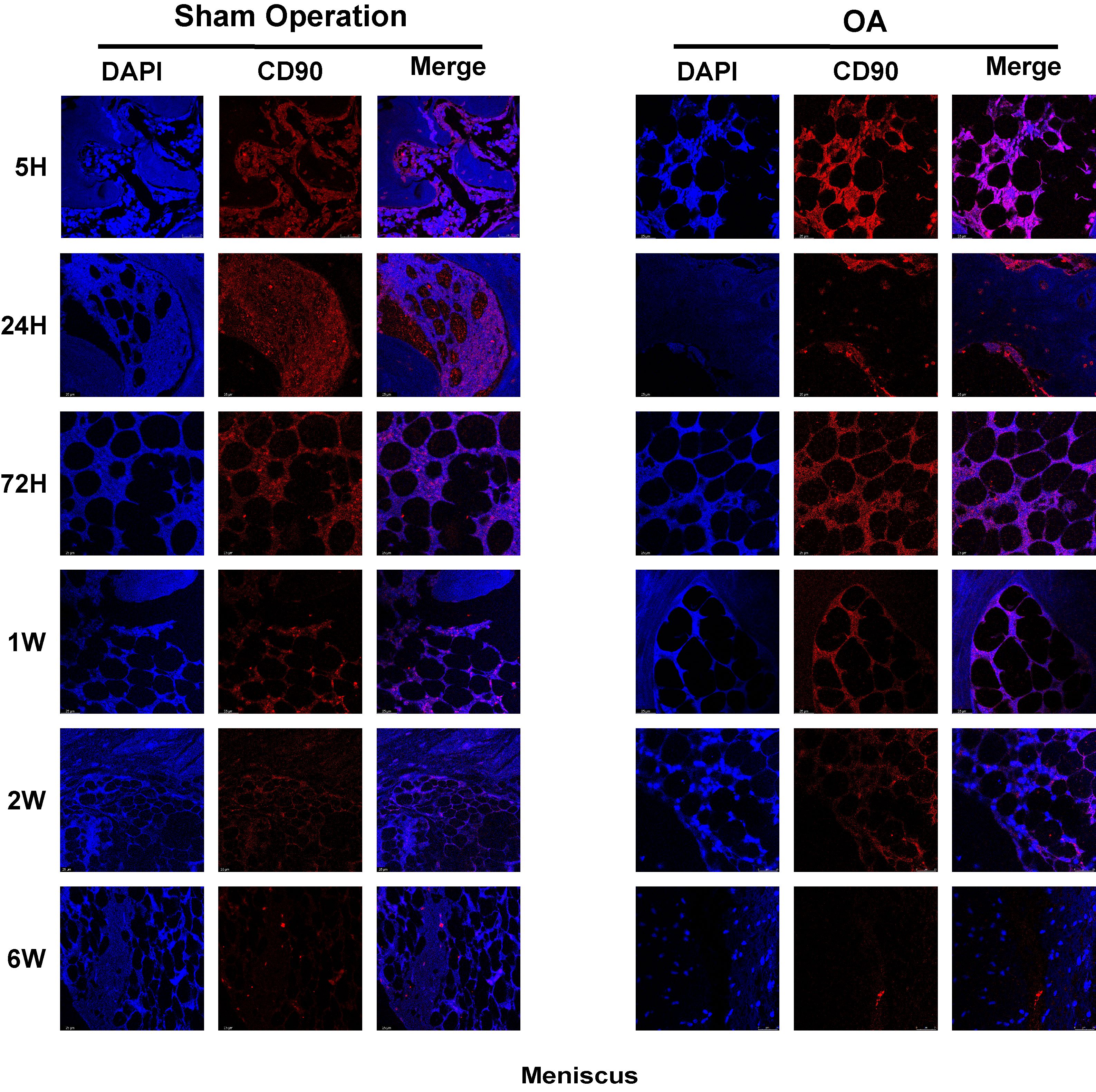

Supplement: Supplementary file 2 [file Image1.jpeg]

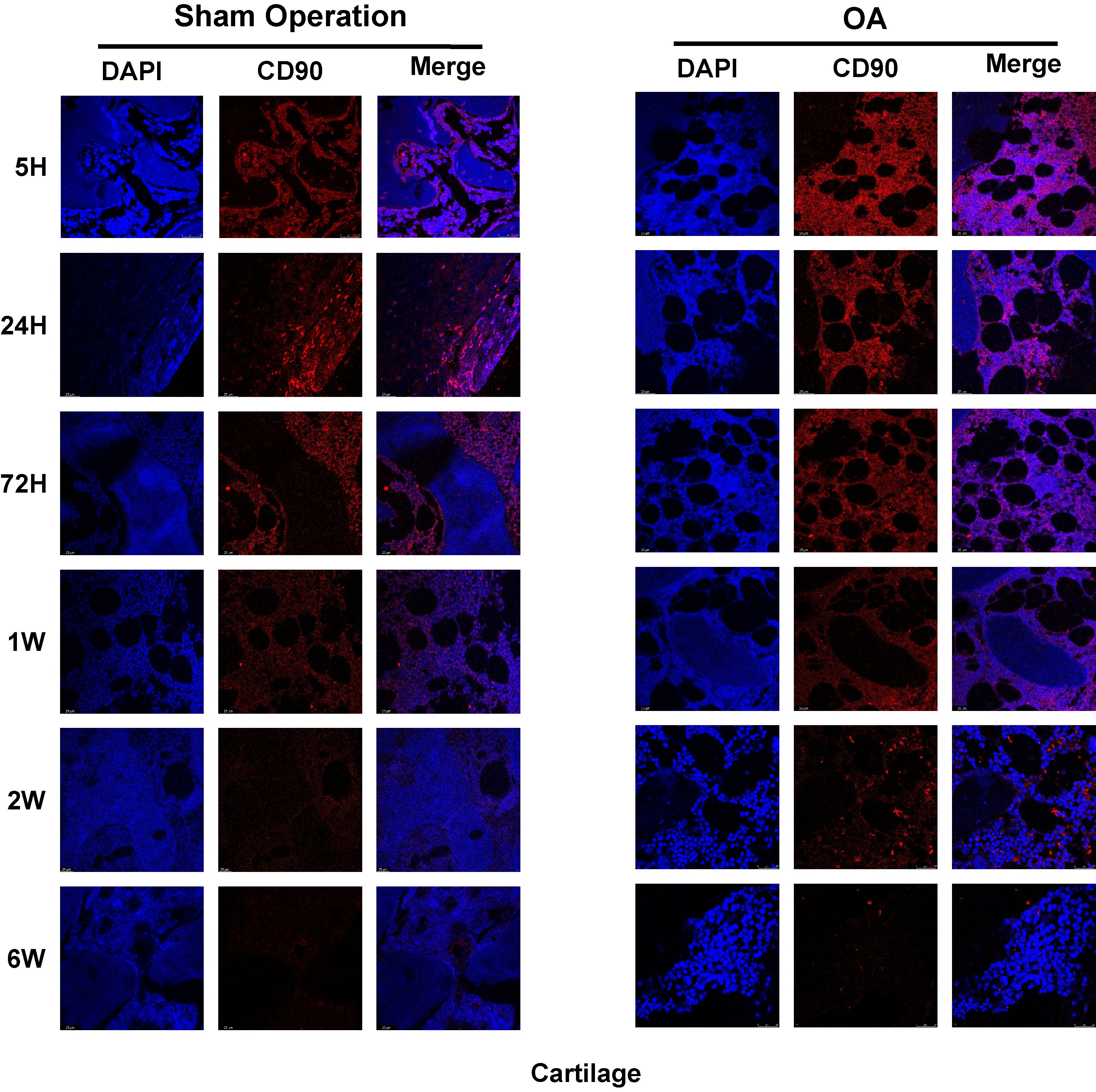

Supplement: Supplementary file 3 [file Image2.jpeg]
